# Effects of treadmill training combined with transcranial direct current stimulation on mobility, motor performance, balance function, and other brain-related outcomes in stroke survivors: a systematic review and meta-analysis

**DOI:** 10.1007/s10072-024-07768-2

**Published:** 2024-09-19

**Authors:** Jibrin Sammani Usman, Thomson Wai-lung Wong, Shamay Sheung Mei Ng

**Affiliations:** Department of Rehabilitation Sciences, The Hong Kong Polytechnic University, Hung Hom, Hong Kong SAR China

**Keywords:** Treadmill training, Mobility, Motor performance, Balance, tDCS, Stroke survivors

## Abstract

**Introduction:**

Treadmill training (TT) is a gait training technique that has commonly been used in neurorehabilitation, and has positive effects on gait, mobility, and related outcomes in stroke survivors. Transcranial direct current stimulation (tDCS) is a non-invasive approach for modulating brain cortex excitability.

**Aim:**

To evaluate the available scientific evidence on the effects of TT combined with tDCS on mobility, motor performance, balance function, and brain-related outcomes in stroke survivors.

**Methods:**

Five databases namely the Cochrane library, PEDro, Web of Science, PubMed, and EMBASE, were searched for relevant studies from inception to March, 2024. Only randomized controlled trials were included, and their methodological quality and risk of bias (ROB) were evaluated using the PEDro scale and Cochrane ROB assessment tool respectively. Qualitative and quantitative syntheses (using fixed effects meta-analysis) were employed to analyze the data.

**Results:**

The results revealed that TT combined with active tDCS had significant beneficial effects on some mobility parameters, some gait spatiotemporal parameters, some gait kinematic parameters, gait endurance, gait ability, and corticomotor excitability in stroke survivors, but no significant difference on gait speed (*P* > 0.05), functional mobility (*P* > 0.05), motor performance (*P* > 0.05), or some balance functions (*P* > 0.05), compared with the control conditions.

**Conclusions:**

TT combined with active tDCS significantly improves some gait/mobility outcomes and corticomotor excitability in stroke survivors.

**Supplementary Information:**

The online version contains supplementary material available at 10.1007/s10072-024-07768-2.

## Introduction

Varying limitations including those in mobility, transfers, walking, navigating stairs and essential daily activities are found in stroke survivors [1]. In addition, in stroke, activities for daily locomotion such as walking on sloped surface, moving around obstacles, climbing up and down the stairs, making turn and changing directions are also impaired [2]. During the gait cycle, stroke survivors typically show altered kinetic and kinematic variables at the hip, knee and ankle joints [3]. The post-stroke gait impairments strongly contribute to overall disability in stroke survivors [4]. Because impairments in walking arise from neuromuscular control deficiency, it is important to better understand the effects of gait training on motor pattern in lower extremity [3]. High intensity treadmill training (HIT), an increasingly popular exercise training in which exercise intensity is maximized by combining short bursts of maximal effort [5], can be used to targets gait difficulties and decreased cardiorespiratory fitness [6].

Treadmill exercises, especially during the acute phase of stroke, have several positive effects, leading to improvements in numerous dimensions [7]. It has been found that turning-based treadmill training (TT) effectively improves functional reorganization of the brain underlying cortico-muscular and cortico-cortical mechanisms, leading to improvements in gait among stroke survivors [8]. Moreover, post-stroke TT has been shown to improve walking distance, but not walking speed and balance, compared with over-ground walking training [9]. TT can improve aerobic capacity more effectively than conventional rehabilitation [2]. Aerobic treadmill exercises have been reported to be used successfully in gait retraining and for enhancing cardiorespiratory fitness [4]. In addition, high intensity speed-based treadmill training has been reported to improve walking speed, walking endurance and quality of life even up to 3 months post intervention [5].

Transcranial direct current stimulation (tDCS) has been established as a non-invasive approach to improving neurorehabilitation [10] involving neuromodulation of the brain cortical areas with possible therapeutic benefits [11]. It consists of applying a low-intensity constant direct current to specific brain areas and is used for modulating neuronal activity and influencing brain function [12]. The weak electrical current induces the regulation of brain cortex activity [13–15]. tDCS can significantly improve walking and lower limb motor functions in stroke survivors [16]. In tDCS, constant low current is delivered between two electrodes, either anodal or cathodal in nature placed on the skull, which results in the modulation of neural excitability [17]. However, a recent systematic review, reported a lack of evidence for the use of tDCS alone for improving motor function, balance and quality of life in stroke survivors; but postulated the beneficial effects of tDCS when combined with other interventions [18].

To the extent of what we know, and given the background provided above, there has not been a systematic review on the effects of TT combined with tDCS on the outcomes of interest. Hence, the purpose of the systematic review and meta-analysis was to determine the scientific evidence on the effects of TT combined with tDCS on mobility, motor performance, balance function, and other brain related outcomes in stroke survivors.

## Methods

**Fig. 1:**
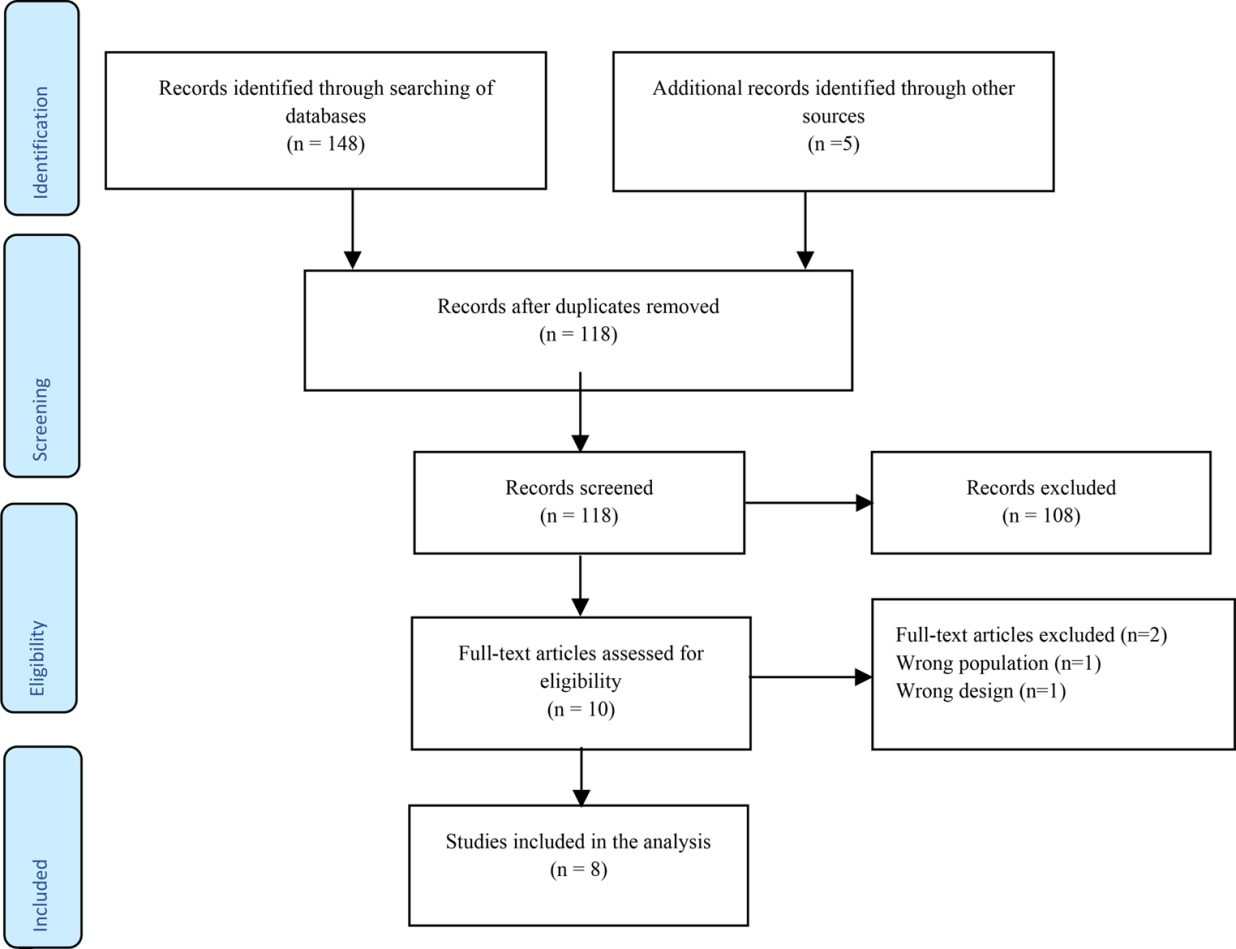
PRISMA flow chart of the review process

The Preferred Reporting Items for Systematic reviews and Meta-Analyses (PRISMA) guide was followed in carrying out the review (Fig. 1), and the review has been registered in PROSPERO (registration number: CRD42024534889). The PICOS strategy was used to construct the following research question: What is the effect of TT combined with active tDCS (I = intervention) on mobility, motor performance, balance function and other brain-related outcomes (O = outcome) in stroke survivors (P = population), when compared with TT alone or combined with sham tDCS (C = comparison) in randomized controlled trials (RCTs) (S = study design)?

### Strategy for search

Five online databases, namely the Cochrane library, PEDro, Web of Science, PubMed, and EMBASE online data bases were searched for relevant studies from inception to March; 2024. The search strategy employed in the data bases was produced using the PICOS strategy. All of the data bases were searched in accordance with their requirements. Details about the search approach employed in the online data bases are presented in Supplementary Material 1. In addition, there was a manual search of the reference lists of the relevant studies and reviews. Similarly, studies obtained from the general literature search were involved in the review. The studies found were imported into the reference manager and duplicates were removed, whereas the results that remained undergone additional screenings in the title, abstract and full text. The search was conducted independently by one author (JSU), and verified by the other two authors (TWLW and SSMN).

### Criteria for eligibility

Studies that met the following inclusion criteria were included in the review: studies (i) performed in human stroke survivors and published in English in peer reviewed journals, (ii) that reported the effects of TT combined with tDCS, and (iii) that had a cross-over or parallel RCT design with full text obtainable. Abstracts presented in conferences, theses, and review studies were left out.

### Data extraction and selection of studies

After expunging the duplicate studies, two authors (JSU and TWLW) independently screened the titles and abstracts of the remaining studies in line with the review selection criteria. Any disagreement between the two authors regarding the eligibility or inclusion of a study were resolved by consulting the other author (SSMN). For full text screening, the full-texts of relevant studies have been obtained, and the following data were extracted from each study: author and publication year, design, sample size, sex, age, clinical characteristics of the population (chronicity, stroke duration, stroke type, and affected side), intervention group(s), outcomes, TT protocols, evaluation periods, parameters for stimulation (type, site, intensity, period, size of electrode, number of stimulation sessions, and side effects), results and conclusions. The extracted data was inputted into Microsoft Excel.

### Assessment of methodological quality (MQ) and risk of bias (ROB)

The MQ of the studies included was appraised using the PEDro scale. The scale has eleven items used to score the MQ of the studies [19], the 11 items assess the internal validity and statistical reporting of the study, excluding initial item that deals with eligibility criteria and is excluded in computing the total score [20]. Thus, the internal validity consist of 10 items that are rated as 1 or 0 signifying Yes (present) and No (absent) respectively [20]. Total scores of 0–3, 4–5, 6–8, and 9–10 are considered to indicate poor, fair, good and excellent MQ respectively [21, 22].

**Fig. 2:**
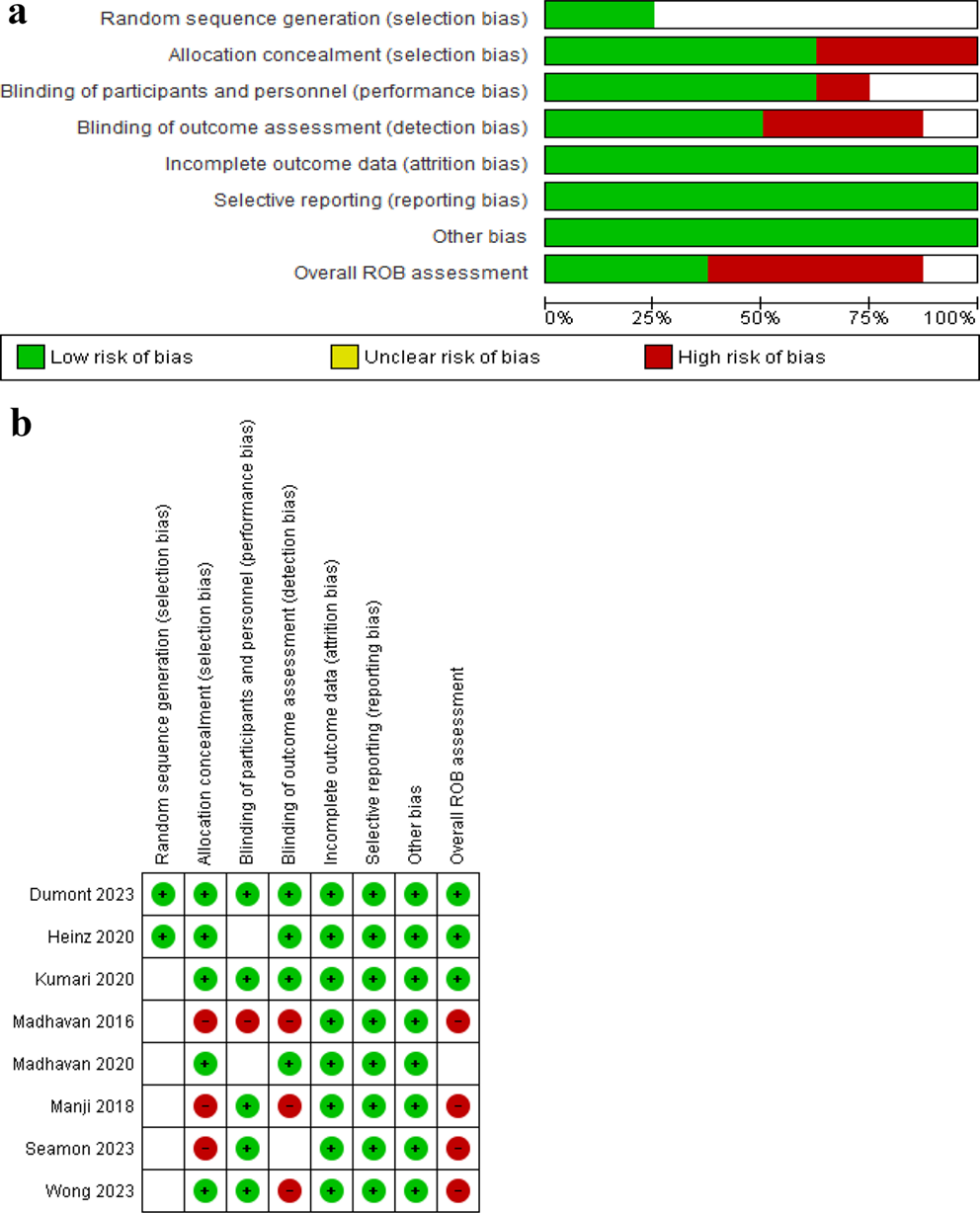
**a** Risk of bias graph for included studies. **b** Risk of bias summary for included studies

The Cochrane ROB tool for RCTs was applied to appraise the ROB in the studies involved in the review (Fig. 2a & b). The tool provide three decision (unclear, high or low) regarding the ROB for individual items in the following domains of bias in: selection, reporting, performance, detection, attrition and other sources [23]. Each study receives an overall judgement (unclear, high, or low) based on the total score obtained by summing the individual domain scores. Two authors (JSU and TWLW) conducted the ROB evaluation independently. Any disagreements were resolved by consulting the other author (SSMN).

### Data analysis

Qualitative and quantitative syntheses were applied to analyze the data that was retrieved. In the qualitative synthesis, the MQ, ROB, and characteristics of the relevant studies were explained. The quantitative synthesis involved a fixed effects meta-analysis (FEMA) of the mean and standard deviation values of the outcomes post-intervention and the sample sizes of the relevant studies. For outcomes for which meta-analyses could not be performed, e.g., because less than two studies reported them, the mean and standard deviation values were used to compute the effect sizes (ESs) for these outcomes. The Cochrane GRADE approach was used to synthesize and assess the certainty of the evidence.

## Results

### Qualitative synthesis of the results

In the qualitative synthesis, the data were synthesized narratively.

### Identification and selection of eligible studies

In total, 153 relevant studies were identified by the searching the online databases and added sources. Subsequent to the removal of duplicates (35), 118 studies left. Title and abstract screening; led to the exclusion of 108 studies for not conforming with the eligibility criteria. Subsequently, the full-texts of the remaining 10 studies were assessed against the eligibility criteria for the review. Eight (8) studies were found to be suitable and were involved in the review. All of the included studies were RCTs with cross-over or parallel design. Figure 1 shows the procedure of identification and selection of studies in line with the PRISMA guidelines.

### MQ of the relevant included studies

Of the eight included studies, four had good and, four had excellent MQ. Regarding the domains of the PEDro scale, all of the included specified the eligibility criteria, conducted random allocation, had groups that are similar at baseline in terms of important prognostic indicators, measured the key outcomes from majority of participants initially allocated to groups, had all subjects whom outcome measures were available received intervention as allocated, and reported between group comparisons and provides point measures and measures of variability for key outcomes. In all the studies except three [24–26], there was allocation concealments. All studies had subjects blinding except in two studies [24, 27], half of the studies had experimenter blinding and half have not, and all the studies except three [24–26] adopted outcome assessor blinding. Table 1 provides the methodological details of the included studies.

**Table 1:** Methodological quality of the relevant included studies according PEdro criteria

**Study/Author (year)**	**Item 1**	**Item 2**	**Item 3**	**Item 4**	**Item 5**	**Item 6**	**Item 7**	**Item 8**	**Item 9**	**Item 10**	**Item 11**	**Total Score**	**Overall** **Quality** **assessment**
Dumont (2023)	Yes	Yes	Yes	Yes	Yes	Yes	Yes	Yes	Yes	Yes	Yes	10	Excellent quality
Heinz (2020)	Yes	Yes	Yes	Yes	Yes	No	Yes	Yes	Yes	Yes	Yes	9	Excellent quality
Kumari (2020)	Yes	Yes	Yes	Yes	Yes	Yes	Yes	Yes	Yes	Yes	Yes	10	Excellent quality
Madhavan (2016)	Yes	Yes	No	Yes	No	No	No	Yes	Yes	Yes	Yes	6	Good quality
Madhavan (2020)	Yes	Yes	Yes	Yes	No	No	Yes	Yes	Yes	Yes	Yes	8	Good quality
Manji (2018)	Yes	Yes	No	Yes	Yes	Yes	No	Yes	Yes	Yes	Yes	8	Good quality
Seamon (2023)	Yes	Yes	No	Yes	Yes	Yes	No	Yes	Yes	Yes	Yes	8	Good quality
Wong (2023)	Yes	Yes	Yes	Yes	Yes	No	Yes	Yes	Yes	Yes	Yes	9	Excellent quality

**Item 1**: eligibility criteria specified; **Item 2**: subjects were randomly allocated to groups(in a crossover study, subjects were randomly allocated an order in which treatments were received); **Item 3**: Allocation was concealed; **Item 4**: the groups were similar at baseline regarding the most important prognostic indicators; **Item 5**: there was blinding of all subjects; **Item 6**: there was blinding of all therapists who administered the therapy; **Item 7**: there was blinding of all assessors who measured at least one key outcome; **Item 8**: Measures of at least one key outcome were obtained from more than 85% of the subjects initially allocated to groups; **Item 9**: all subjects for whom outcome measures were available received the treatment or control condition as allocated or, where this was not the case, data for at least one key outcome was analyzed by “intention to treat”; **Item 10**: the results of between-group statistical comparisons are reported for at least one key outcome; **Item 11**: The study provides both point measures and measures of variability for at least one key outcome

### ROB in the relevant included studies

All of the studies had a low ROB in the domains of attrition, reporting and other biases, while half of the studies had a low and the other half had a high ROB in detection bias; In addition, a majority of the studies had a low ROB in performance bias and selection bias (allocation concealment), and most of the studies had an unclear ROB in selection bias (random sequence generation). Figure 2a and b provides the ROB assessment results of the included studies.

### Characteristics of the relevant included studies

All of the studies included chronic stroke survivors except one [25] that included subacute stroke survivors. Four studies employed a parallel RCT design, and the other four adopted a cross-over RCT design. In total, 227 subjects participated in the included studies, of whom 146 were male. The mean age range of the participants was 55.43 to 67.25 years, and the mean stroke duration ranged from 25.50 to 121.25 months. Regarding the stroke type, 122 participants had ischemic stroke, while 73 had hemorrhagic stroke. One study [26] did not report the stroke type. The affected side was right in 93 participants, and one study [25] did not report the affected side. Regarding the tDCS type, all of the studies used real/active tDCS and sham tDCS combined with TT. In terms of outcomes assessment, all of the included studies, performed pre and post intervention outcome assessments. Table 2 presents the characteristics of the included studies.

**Table 2:** Characteristics of relevant included studies

**Author (Year)**	**Design**	***N*** **Sex**	**Age (years)** **Mean ± SD**	**Clinical Characteristics of the patient population**	**Intervention(s)/ Groups**	**Outcomes**	**Treadmill training protocols** **Evaluation period**
Dumont (2023) [28]	RCT-P	28 13 M,11 F	EG: 58.5 ± 10.04 CG: 58.4 ± 11.44	Chronicity: Chronic Stroke duration (m): EG:39.2(20.2); CG: 25.5 (19.4) Stroke type: IS = 15; HM = 9 Side affected: *R* = 10; L = 14	EG: a-tDCS + TT CG: Sham + TT	Gait speed (GS), spatiotemporal and kinematic variables, and functional capacity	20 min of TT with no BWS, 5 times/week for 2 weeks Pre and post
Heinz (2020) [29]	RCT-CR	12 8 M,4 F	59 ± 7.00	Chronicity: Chronic Stroke duration (m): 65.45 ± 54.86 Stroke type: IS = 9; HM = 3 Side affected: *R* = 7; L = 5	ECN: a-tDCS + TT CCN: Sham + TT	Heart rate variability (HRV), variability in systolic blood pressure (VSBP),	20 min of TT Pre and post tDCS and during the exercise
Kumari (2020) [30]	RCT-P	4 2 M,2 F	67.25	Chronicity: Chronic Stroke duration (m): 121.25 Stroke type: IS = 2; HM = 2 Side affected: *R* = 2; L = 2	EG: c-tDCS + TT CG: Sham + TT	Motor learning, step length symmetry, walking speed, step length	15 min of TT Pre and post intervention
Madhavan (2016) [24]	RCT-CR	11 4 M,7 F	58 ± 2.7	Chronicity: Chronic Stroke duration (y): 9 ± 1.8 Stroke type: IS = 7; HM = 4 Side affected: *R* = 7; L = 4	ECN: e-tDCS + HIIT CCN: HIIT alone	GS, corticomotor excitability (CME), rating of perceived exertion, Heart rate	15 min of TT Prior and at the end
Madhavan (2020) [27]	RCT-P	81 55 M,26 F	A. tDCS + HISST: 58 ± 11 B. CG: 58 ± 10 C. AMT + HISST: 60 ± 9 D. tDCS + AMT + HISST: 59 ± 9	Chronicity: Chronic Stroke duration (y): A (4.3 ± 3.6), B (6.1 ± 4.2), C (5.6 ± 3.6) and D (5.9 ± 5.6) Stroke type: IS = 53; HM = 26 Side affected: *R* = 37; L = 44	(A) tDCS + HISST (B) G HISST only G C. AMT + HISST G D. tDCS + AMT + HISST G	Walking speed, CME, paretic active motor threshold, paretic MEP, mobility (TUG) balance (BBT, miniBESTest, & ABC), motor function (FMLE), walking endurance (6mWT) & stroke impact (SIS)	40 min of TT, 3 sessions per week for 4 weeks Pre post and 3 month follow up
Manji (2018) [25]	RCT-CR	30 21 M,9 F	EG: 62.2 ± 10.10 CG: 63.7 ± 11.0	Chronicity: Subacute Stroke duration (m): EG = 4.48 ± 1.86, CG = 4.99 ± 0.81 Stroke type: IS = 17; HM = 13	EG: a-tDCS + BWSTT CG: Sham + BWSTT	Walking speed (10 MWT), Mobility (TUG, POMA), balance (TCT &TIS), motor function (FMLE)	20 min of BWSTT, once a day for a week Pretest, posttest 1 and 2
Seamon (2023) [26]	RCT-CR	16 11 M,5 F	59 ± 11.5 (30–77)	Chronicity: Chronic Stroke duration (m): 54.56 ± 76.5 (10–325) Stroke type: Side affected: *R* = 9; L = 7	ECN: a-tDCS + TT ICN: ct-tDCS + TT DCN: ECN + ICN Sham: Sham + TT	Gait speed, paretic step ratio, paretic propulsion, walking performance (PF), motor function (FMLE), balance (BBS), DGI, & modules, muscle activation (EMG)	15 min of TT Pre and post
Wong (2023) [31]	RCT-P	45 32 M,10 F	A. b-tDCS + TT: 55.43 ± 5.39 B. ct-tDCS + TT: 60.64 ± 11.3 C. Sham + TT: 64.05 ± 9.4	Chronicity: Chronic Stroke duration (m): A (70.71 ± 48.0), B (54.43 ± 38.7), C (72.57 ± 57.10) Stroke type: IS = 26, HM = 16 Side affected: *R* = 28; L = 14	G1: tDCS + TT G2: ct-tDCS + TT G3: Sham + TT	Dual-task walking (DTW) PF, walking PF, gait spatiotemporal parameters, contralesional cortical activity (RMT, MEP, & SICI), motor control of lower extremity (FMLE), cognitive DTW PF, & motor DTW PF.	30 min of TT, 3 sessions per week for 4 weeks Pre and post

ABC: Activity specific balance confidence scale; AMT: ankle motor tracking; a-tDCS: Anodal transcranial direct current stimulation; BBT: Berg balance test; b-tDCS: Bilateral transcranial direct current stimulation; BWST: Body weight supported treadmill training; ct-DCS: Cathodal transcranial direct current stimulation; CG: Control group; CN: Condition; CME: corticomotor excitability; CRO: Cross-over; DCN: dual condition; DGI: Dynamic gait index; DTW: Dual-task walking; ECN: excitatory condition; EG: Experimental group; EMG: Electromyogram; F: Female; FMLE: Fugl-Meyer motor assessment (lower extremity); G: Group; GS: Gait speed; HIIT; High intensity treadmill training; HISST: High-intensity speed-based treadmill training; HM: Hemorrhagic; HRV: Heart rate variability, ICN: inhibitory condition; IS: Ischemic; L: Left; M: Male; m: months; MEP: motor evoked potentials; N: Total sample size; PF: Performance; POMA: Performance Oriented Mobility Assessment; R: Right; RCT-P: Parallel Randomize controlled trial; RMT: resting motor threshold; SD: Standard deviation; SICI: short-interval intracortical inhibition; SIS: Stroke impact scale; TCT: Trunk control test; tDCS: Transcranial direct current stimulation; TIS: Trunk impairment scale; TT: Treadmill training; TUG: Time up and go test; VSBP: variability in systolic blood pressure; y: years; 6mWT: six minute walk test; 10MWT: 10 m walk test

### **Described outcomes**

#### Gait speed

Eight studies reported the gait speed of the participants [24–31].

#### Mobility

Two studies reported functional mobility [25, 27], and one study reported mobility [25]. Walking performance was reported in two studies [26, 31].

#### Motor function

Four studies reported motor function [25–27, 31].

#### Functional capacity

Two studies reported on functional capacity [27, 28].

#### Other gait spatiotemporal and related parameters

One study [28] reported stance phase, double limb support time (DBST), and step width, and one study [31] reported cadence, and step time. Step length was reported in three studies [28, 30, 31], and step length symmetry was reported in two studies [26, 30]. One study [26] reported the paretic step ratio and paretic propulsion, and one study [28] reported kinematic parameters.align=“left”

#### Balance-related parameters

One study reported balance performance and balance confidence [27] while another one reported trunk control [25].

#### Other brain-related parameters

Corticomotor excitability was reported in three studies [24, 27, 31].

#### Cardiovascular related parameters

The heart rate and/or its variability was reported in two studies [24, 29], while blood pressure was reported in three studies [24, 28, 29].

### tDCS procedures

#### Types of stimulation

All of the included studies except one [31] conducted anodal tDCS, while one study [26] conducted cathodal and dual stimulations in addition to anodal stimulation. One study reported bilateral and cathodal stimulations [31].

#### Site of electrodes during tDCS

The anode electrode placement on the leg motor area (M1)/motor cortex was reported in all of the included studies except one [29] in which the placement was lateral to the inion. Cathode electrode placement on the orbital region was reported in four studies [24, 27, 28, 31], while the other studies reported cathode placement on the middle deltoid muscle [29], ipsilateral buccinator muscle [30], inion [25], ipsilateral shoulder [26], and contralesional M1 [26, 31].

#### Intensity and duration of stimulation

Five studies applied a tDCS intensity of 2.0 mA [26, 28–31], and three studies applied a tDCS intensity of 1.0 mA [24, 25, 27]. Regarding stimulation duration, five studies reported a duration of 20 min [25, 26, 28, 29, 31], two studies reported 15 min [24, 27], and one study reported 10 min [30].

#### Size of electrodes

With regards to the anode/active electrode size, two studies [29, 30] reported 5 × 5 cm^2^, one [25] reported 5 × 5 cm, while one study each reported 8cm^2^ [24], 5 × 2.5 cm [27], 1.75cm^2^ [26] and 35cm^2^ [31].

#### Number of stimulation sessions

Concerning number of stimulation sessions, when the studies were reviewed, two studies conducted twelve sessions [27, 31]. Additionally, one study each conducted one [29], two [24], three [30] four [26], seven [25], and ten [28] sessions.

#### Side effects of tDCS

Two studies reported side effects [27, 30]. In two studies [24, 31] there participants reported no adverse/side effects, while in another study [28], no detail information was reported about the adverse/side effects. However, three studies [25, 26, 29] reported no information on adverse/side effects linked to tDCS. Table 3 provides the details of the tDCS protocols used in the included studies.

**Table 3:** tDCS procedures and results of the relevant studies

**Studies**	**Stimulation**					**Findings**	
**Author (Year)**	**Type**	**Site (Based on 10–20 EEG electrode placement and nomenclature system)**	**Intensity (mA) Period (minutes)**	**Size of electrode** **No. of stimulation session**	**Side effects**	**Results**	**Conclusion**
Dumont (2023) [28]	Anodal	AN: Primary motor cortex (C3 or c4) CT: Contralateral supraorbital region	2 mA 20 min	5cm^2^ 10	NI	There is significant difference in EG in kinematic gait parameters such as PT-IC, HAA-ROM, KMSW, K-ROM, AMSt, AMSw, and A-ROMst. But no significant difference in gait spatiotemporal variables between groups	tDCS combined with simultaneous TT did not have significant effect on gait spatiotemporal parameters, but has significant effect on gait kinematic parameters.
Heinz (2020) [29]	Anodal	AN: Left temporal cortex CT: Middle deltoid muscle	2 mA 20 min	5 × 5cm^2^ 1	NR	There was no difference in VSBP and HRV between groups compared to baseline data.	tDCS does not generate immediate effects on HRV and VSBP except larger participation in parasympathetic regulation in active tDCS group.
Kumari (2020) [30]	Anodal	AN: Lateral to the inion CT: Ipsilateral buccinator	2 mA 10 min	5 × 5cm^2^ 3	Cheek twitching and mild cheek tingling	The result showed that the planned RCT protocol and ctDCS-SBTT intervention are not feasible.	The study showed substantial variability in the direction of step length asymmetry affecting the recruitment and delivery of SBTT.
Madhavan (2016) [24]	Anodal	AN: Hotspot of paretic leg M1 CT: Forehead above the contralateral orbit	1 mA 15 min	AN: 8cm^2^ CT: 35cm^2^ 2	No	e-tDCS + HIIT induced an increase in CME of the paretic TA, and corresponding increase in CME of the nonparetic TA. Both HIIT and e-tDCS showed trend towards improved overground gait speed.	Single session of HIIT has the potential to exacerbate suppressed corticomotor excitability of paretic lower limb muscle representations in some individuals with stroke.
Madhavan (2020) [27]	Anodal	AN: Leg representation of ipsilesional motor cortex (M1) CT: Contralateral supraorbital region	1 mA 15 min	AN: 5 × 2.5 cm CT: 4.5 × 5.5 cm 12	Minimal	Four weeks of HISTT result in improvement in walking speed and endurance, which were maintained partially 3 months after training.	HISTT is a feasible and effective gait training paradigm for individuals with chronic stroke.
Manji (2018) [25]	Anodal	AN: 3.5 cm anterior to Cz CT: Over the inion	1 mA 20 min	5 × 5 cm 7	NR	Anodal tDCS over the SMA may enhance improvement in gait ability in combination with BWSTT in hemiparetic stroke survivors.	Therapy with tDCS over the SMA combined with BWSTT contributes to an improvement in gait ability in stroke.
Seamon (2023) [26]	Anodal Cathodal Dual	ECN: AC (AN): Ipsilesional leg M1 area), RF: Ipsilateral shoulder ICN: AC (CT): Contralesional leg M1 area, RF: Ipsilateral shoulder DCN: Simultaneous application of montages in ECN and ICN To target both leg M1 areas.	2 mA 20 min	1.75cm^2^ 4	NR	No group main effects for any of the tDCS electrode montages compared with sham stimulation on walking performance immediately post one session of tDCS.	A single tDCS session may affect clinical and biomechanical walking performance, but effects seem to be dependent on individual response variability to different electrode montages.
Wong (2023) [31]	Bilateral Cathodal	b-tDCS + TT G: AN: Ipsilesional M1, CT: contralateral M1, ct-tDCS + TT G: AN: contralateral supra orbital ridge, CT: contralesional M1 Sham + TT G: as obtained in ct-tDCS + TT G	2 mA 20 min	35cm^2^ 12	No	Cathodal tDCS followed by TT can lead to better effects on the CDTW, speed, cadence, and step time of the paretic leg than TT alone. It also significantly increased inhibition and decrease the excitability of the contralesional M1 more than TT alone.	There were superior positive effects of cathodal tDCS followed by TT on CDTW performance and contralesional cortical activity than TT alone.

AC: Active; AN: Anode; AMSt: maximum angle of ankle dorsiflexion in stance phase; AMSw: maximum angle of ankle dorsiflexion in swing phase; A-ROMst: range of motion of ankle in stance phase; b-tDCS: bilateral transcranial direct current stimulation; CT: Cathode; tDCS: Transcranial direct current stimulation; ct-tDCS: Cathodal transcranial direct current stimulation; CDTW: Cognitive-dual-task walking; CME: Corticomotor excitability; DCN: dual condition.; e-tDCS: anodal tDCS enhanced with a skill acquisition task; ECN: excitatory condition; EG: Experimental group; G: Group; HAA: hip abduction/adduction; HIIT; High intensity treadmill training; HISST: High-intensity speed-based treadmill training; HRV: Heart rate variability; IC: initial contact; ICN: inhibitory condition; KMSW: maximum angle of knee flexion in swing phase; M1: Primary motor cortex; mA: milli ampere; NI: No detail information about the adverse effects; No: No adverse effect reported by participants; NR: No information reported in the study; PT-IC: Kinematic pelvis variables; RCT: Randomized controlled trial; ROM: Range of motion; SBTT: split-belt treadmill training; SMA: Supplementary motor area; TA: tibialis anterior; TT: Treadmill training; VSBP: variability in systolic blood pressure

### Qualitative synthesis, and quantitative synthesis

#### Effects of the interventions

##### Effects on gait speed

**Fig. 3:**
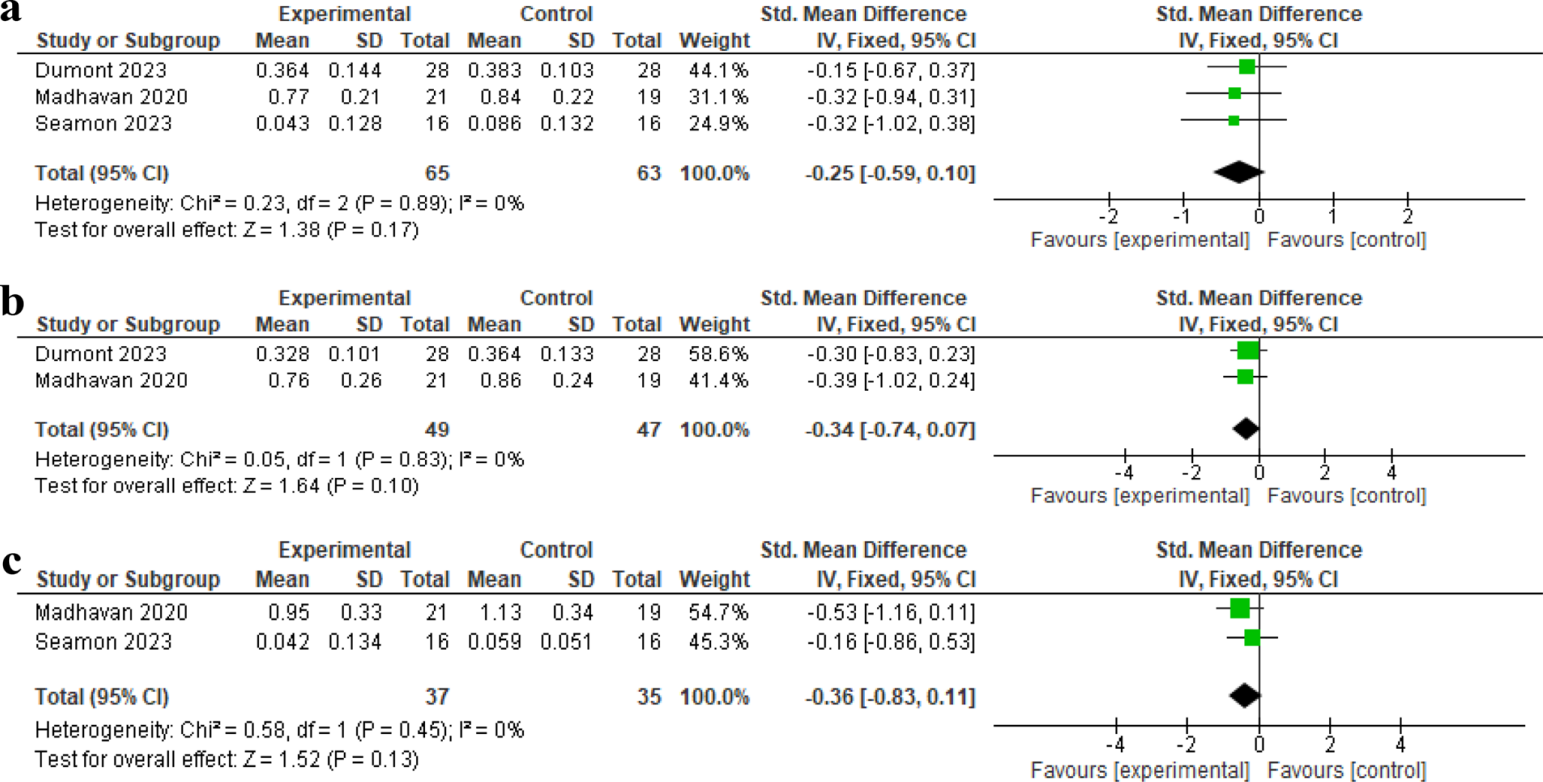
**a** Effect of TT combined with tDCS on comfortable gait speed at initial sessions. **b** Effect of TT combined with tDCS on comfortable gait speed at follow-up sessions. **c** Effect of TT combined with tDCS on fastest walking speed

Three studies evaluated the effect on comfortable gait speed [26–28]. The FEMA results showed no significant difference in comfortable gait speed in the initial sessions post-intervention (standardized mean difference [SMD] = -0.25, 95% confidence interval [CI] -0.59 to 0.10, *P* = 0.17) (Fig. 3a) (with low certainty of evidence downgraded due to ROB and imprecision), comfortable gait speed in the follow-up sessions post-intervention (SMD = -0.34, 95% CI -0.74 to 0.07, *P* = 0.10) (Fig. 3b) (with moderate certainty of evidence downgraded due to imprecision), or fastest walking speed (SMD=-0.36, 95%CI -0.83 to 0.11, *P* = 0.13) (Fig. 3c) (with low certainty of evidence downgraded due to imprecision and high ROB); among stroke survivors between the experimental and control groups. However, Speed-based high-intensity interval training (HISTT) has been found to improve gait speed in stroke survivors [27]. Additionally, TT combined with active tDCS significantly improved walking speed [25, 28], and cognitive dual task walking speed [31]. Also TT combined with active tDCS shows trend toward improving gait speed [24]. There was also positive improvement in walking speed especially in the TT with sham tDCS group post intervention [30].

##### Effects on mobility

The ES showed no significant difference in functional mobility (mean difference [MD] = 2.80, 95% CI -0.91 to 6.51, *P* = 0.14) [27], (MD = -3.10, 95% CI -6.87 to 0.67, *P* = 0.11) [25] between the experimental and control groups. For other outcomes, the calculated ES revealed a significant difference in performance-oriented mobility (MD = 0.56, 0.04 to 1.07, *P* = 0.03) between the experimental and control groups [25]. Anodal tDCS over the supplementary motor area combined with BWSTT was found to possibly improve gait ability in stroke survivors [25]. Also, TT combined with active tDCS has superior positive effect on cognitive dual task walking performance [31]. Also, it was found that clinical and biomechanical walking performance may be affected with the intervention [26].

##### Effects on motor performance

The calculated ES revealed no significant difference in motor function (MD = 1.20, 95% CI -1.37 to 3.77, *P* = 0.36) [27], (MD = -1.50, 95% CI -4.12 to 1.12, *P* = 0.26) [25] between experimental and control group in stroke survivors. However, TT combined with active tDCS significantly improved lower extremity motor control [31].

##### Effects on functional capacity

The calculated ES revealed no significant difference in the 6-minute walk test score (MD = -0.143, *P* = 0.18) between the experimental and control groups. HISTT was found to improve endurance in stroke survivors [27].

##### Effects on other gait spatiotemporal and related parameters

The calculated ES revealed no significant difference in the stance phase (after one session) (SMD = 0.21, *P* = 0.43), stance phase (after 10 sessions) (SMD = 0.34, *P* = 0.20), stance phase (at the one-month follow up) (SMD = -0.10, *P* = 0.70), between the experimental and control groups. Furthermore, the calculated ES revealed a significant difference in DBST (after one session) (MD = 0.56, 0.04 to 1.07, *P* = 0.006) between the experimental and control groups, but no significant difference in DBST (after 10 session) (MD = 0.38, *P* = 0.16) and DBST (at the one-month follow up) (MD = 0.02, *P* = 0.95) between the experimental and control groups. Similarly, no significant difference was observed in the step length (*P* > 0.05), 10-meter walk test score (*P* > 0.05), paretic step ratio (*P* > 0.05) and paretic propulsion (*P* > 0.05), but significant differences were observed in the step width (SMD = 0.93, 0.38 to 1.49, *P* = 0.0010) and stroke impact (SMD = -0.82, -1.46 to -0.18, *P* = 0.01) between the experimental and control groups. Additionally, cathodal tDCS followed by TT can result to better effects on the cognitive dual-task walking (CDTW), speed, cadence, and step time of the paretic leg than TT alone [31]. One study [28] revealed a significant improvement in gait kinematic parameters.

##### Effects on balance-related parameters

The calculated ES revealed no significant difference in trunk control (MD = 0.05, *P* = 0.84), balance performance (*P* > 0.05), or balance confidence (MD = -0.12, *P* = 0.71) between the experimental and control groups. Also, although both Berg balance test and mini-BEST test scores shows time effect, and the scores were higher at post assessment and 3 month follow up, but no group effect or group X time interaction with the interventions [27].

##### Effects on other brain-related parameters

The calculated ES revealed no significant difference in corticomotor excitability (CME; *P* > 0.05) [27]. In addition, anodal tDCS enhanced with ankle-tracking (e-tDCS) + HIIT induced an increase in the CME of the paretic tibialis anterior (TA) with a corresponding increase in non-paretic CME [24]. Cathodal tDCS followed by TT significantly increased the inhibition; and decreased the excitability of the contralesional M1 more than TT alone [31].

##### Effect on cardiovascular-related parameters

The calculated ES revealed significant difference in the variance of heart rate variability (HRV) post-tDCS in favor of the experimental group (MD = -370.91, *P* = 0.04) [29]. But, there was reported no significant between group differences in systolic blood pressure and heart rate variabilities in the evaluation periods [29].

## Discussion

The key objective of this study was to evaluate the scientific evidence on the effects of TT combined with tDCS on mobility, motor performance, balance function and other brain-related outcomes in stroke survivors. The major findings of the quantitative synthesis revealed that TT combined with active tDCS had a significant impact on DBST, step width, performance-oriented mobility, and stroke impact among stroke survivors. However, no significant differences were observed in other spatiotemporal parameters such as gait speed, functional mobility, motor performance, and some balance function outcomes between the experimental and control groups. The qualitative analysis findings showed that the interventions in the experimental group led to significant improvements in gait ability, gait endurance, some gait kinematic parameters, CDTW, gait speed, cadence, step time, and corticomotor excitability in stroke survivors compared with the control group. The review included eight RCTs. Qualitative and quantitative syntheses were used for data analysis.

### Quantitative findings

The results revealed that in stroke survivors, TT combined with active tDCS led to significantly better step width, performance-oriented mobility, and DBST than the control interventions. Improvements in the above parameters may go a long way in improving the balance of stroke survivors. These improvements may have occurred possibly because of the nature of the task involved in TT which has to be executed with lower extremities, and all the improved parameters involved the lower extremities. Such may be supported by the previous finding that TT is effective in improving some mobility and gait parameters posts-stroke [32]. In addition, the additional effect of active tDCS may have contributed to the improvements. Although improvements were found in the above parameters, the findings for the outcomes were from few studies involved in the meta-analysis, some findings on some outcomes were only based on qualitative synthesis of some of the RCTs and the calculated effect sizes. In addition, even though the results tended to favoring the experimental group, the results of meta-analysis showed no significant difference between the effects of TT combined with active tDCS and those of TT combined with sham on other spatiotemporal parameters such as gait speed, functional mobility, motor performance, and some balance function outcomes. Hence, further research is warranted to support the existing evidence; because the few studies included in the meta-analysis had limited sample sizes and some methodological flaws. The above-mentioned evidences limit the certainty of the conclusions drawn and calls for more research to obtain more robust conclusions.

### Qualitative findings

The qualitative analysis results revealed that the interventions in the experimental group led to significant improvements in gait ability, gait endurance, some gait kinematic parameters, CDTW, gait speed, cadence, step time, lower extremity motor control and corticomotor excitability in stroke survivors compared with the control groups. This implies that TT combined with tDCS enhances gait/mobility and corticomotor excitability. These findings were supported by three studies [5, 8, 32]. It is vital to note that the findings of improvements in some of the parameters should be interpreted cautiously because the conclusions were derived considering the results of qualitative synthesis and the calculated ESs from the studies; hence, future studies are needed to confirm the findings.

Some of the studies revealed no significant differences in other spatiotemporal parameters, such as gait speed, functional mobility, motor performance, and some balance function outcomes between the experimental and control groups. This finding further supports the need for more RCTs in future on the outcomes of interest; to establish firmer conclusions.

Although; the studies included in the analysis were of good or excellent MQ, some studies did not report adequate data on the outcomes of interest to enable a meta-analysis to be conducted. Hence only ESs were calculated for such outcomes. Additionally, most of the studies included in the review had small sample sizes, and some studies also had an unclear or high ROB in selection and performance bias. These limitations highlight the limited strength of the current evidence; hence, further studies are required to address these limitations and validate the findings.

### Strengths and limitations

The main strengths of this review are adherence to PRISMA guidelines for conducting and reporting the review, searching of relevant online data bases, and use of standardised tools to evaluate the MQ and ROB. The key limitation of the review is that meta-analysis was not performed for some of the outcomes of interest due to inadequate studies or data on these outcomes. Thus, further studies on these outcomes of focus are required. The main limitations of the individual relevant studies were small sample sizes in most, selection and detection bias in many, and performance bias in some of the relevant studies. Thus, caution should be exercised when interpreting the results. Future studies are needed to address these limitations.

## Conclusions

TT combined with active tDCS is beneficial for improving some gait/mobility outcomes and corticomotor excitability in stroke survivors.

## Electronic supplementary material

Below is the link to the electronic supplementary material.

Supplementary Material 1

## Data Availability

Data is available from the corresponding author on reasonable request.
