# Supplementary material for: Effects of treadmill training combined with transcranial direct current stimulation on mobility, motor performance, balance function, and other brain-related outcomes in stroke survivors: a systematic review and meta-analysis
Source: Neurol Sci. 2024 Sep 19;46(1):99–111. doi: 10.1007/s10072-024-07768-2 (PMC11698808; doi:10.1007/s10072-024-07768-2)
Supplement: Supplementary file 1 — Supplementary Material 1 [file 10072_2024_7768_MOESM1_ESM.pdf]

Supplementary Material 1: Search approach used in PubMed data base, and similar search terms were used in other online data bases

((("Treadmill walking" or "Treadmill training" or "Treadmill exercise") AND ((("Transcranial Direct Current Stimulation"[Mesh] OR ("Transcranial direct current stimulation" or tDCS OR ("non invasive brain stimulation"))) AND ((((("Gait"[Mesh] OR "Walking Speed"[Mesh]) OR "Walking"[Mesh]) OR "Lower Extremity"[Mesh]) OR "Postural Balance"[Mesh]) OR ("motor function" or mobility or gait or walking or balance or "lower extremity" or "lower limb"))) AND (((((("Stroke"[Mesh] OR "Stroke, Lacunar"[Mesh] OR "Hemorrhagic Stroke"[Mesh] OR "Embolic Stroke"[Mesh] OR "Thrombotic Stroke"[Mesh] OR "Ischemic Stroke"[Mesh] OR "Infarction, Posterior Cerebral Artery"[Mesh] OR "Brain Stem Infarctions"[Mesh] OR "Infarction, Middle Cerebral Artery"[Mesh] OR "Infarction, Anterior Cerebral Artery"[Mesh]) OR "Cerebrovascular Disorders"[Mesh]) OR "Hemiplegia"[Mesh]) OR "Brain Infarction"[Mesh]) OR "Paralysis"[Mesh]) OR "Paresis"[Mesh])
